# Toward Reproducible Computational Research: An Empirical Analysis of Data and Code Policy Adoption by Journals

**DOI:** 10.1371/journal.pone.0067111

**Published:** 2013-06-21

**Authors:** Victoria Stodden, Peixuan Guo, Zhaokun Ma

**Affiliations:** 1 Department of Statistics, Columbia University, New York City, New York, United States of America; 2 Department of Industrial Engineering and Operations Research, Columbia University, New York City, New York, United States of America; National Institute of Environmental Health Sciences, United States of America

## Abstract

Journal policy on research data and code availability is an important part of the ongoing shift toward publishing reproducible computational science. This article extends the literature by studying journal data sharing policies by year (for both 2011 and 2012) for a referent set of 170 journals. We make a further contribution by evaluating code sharing policies, supplemental materials policies, and open access status for these 170 journals for each of 2011 and 2012. We build a predictive model of open data and code policy adoption as a function of impact factor and publisher and find higher impact journals more likely to have open data and code policies and scientific societies more likely to have open data and code policies than commercial publishers. We also find open data policies tend to lead open code policies, and we find no relationship between open data and code policies and either supplemental material policies or open access journal status. Of the journals in this study, 38% had a data policy, 22% had a code policy, and 66% had a supplemental materials policy as of June 2012. This reflects a striking one year increase of 16% in the number of data policies, a 30% increase in code policies, and a 7% increase in the number of supplemental materials policies. We introduce a new dataset to the community that categorizes data and code sharing, supplemental materials, and open access policies in 2011 and 2012 for these 170 journals.

## Introduction

The journal publication process is a key lever shaping the nature of scholarly communication and promoting the integrity of the scholarly record. The ability to replicate published computational results relies on the availability of the data and code used to generate the findings, and lack of access to such materials is engendering a credibility crisis in the computational sciences [1], [2], [3]. Recent attention has focused on changes needed in scientific publishing and the role of journal open data requirements in fostering scientific reliability [4], [5], [6], [7] but little research has focused on availability of the code needed to replicate computational findings, although this has been associated with high impact publications [8]. A search of four June issues of the Journal of the American Statistical Association, presented in Table 1, shows that authors generally do not provide sufficient information to enable others to access their associated research codes. In this study we document data sharing policies for 170 journals in June 2011 and again in June 2012. In addition, we examine these journals’ code sharing policies and their supplemental materials policies. We seek to understand the nature of these policies, their rate of implementation, and how different journal characteristics may be related to adoption rates.

**Table 1:** Code Availability in the Journal of the American Statistical Association.

JASA June	Computational Articles	Code Publicly Available
1996	9 of 20	0%
2006	33 of 35	9%
2009	32 of 32	16%
2011	29 of 29	21%

There have been numerous calls for data and code release from across the computational sciences [9], [10], [11]. Editorials and commentaries have made the similar appeals [12], [13], [14], [15] and other stakeholders are taking steps to encourage data sharing. Since January 2011 the National Science Foundation (NSF) has required the submission of a 2-page data management plan with every new grant application that outlines plans for the stewardship of the data arising from the funding opportunity (NSF Data Management Plan, Jan. 2011. http://www.nsf.gov/bfa/dias/policy/dmp.jsp). The National Science Foundation and the National Institutes of Health (NIH) both require dataset disclosure and encourage software availability, as seen in the following excerpts from their grant guidelines,

“NSF … expects investigators to share with other researchers, at no more than incremental cost and within a reasonable time, the data, samples, physical collections and other supporting materials created or gathered in the course of the work. It also encourages grantees to share software and inventions or otherwise act to make the innovations they embody widely useful and usable.” (National Science Foundation Grant Guidelines, http://www.nsf.gov/cise/cise_dmp.jsp (2005)) and

NIH (2003): “The NIH endorses the sharing of final research data to serve these and other important scientific goals. The NIH expects and supports the timely release and sharing of final research data from NIH-supported studies for use by other researchers.” For grants over $500,000, a data sharing plan must be included. (National Institutes of Health Grant Guidelines, http://grants.nih.gov/grants/policy/data_sharing/data_sharing_guidance.htm).

Both policies are strongly worded but neither is consistently enforced, and compliance is largely left up to the authors of the papers. A recent proposal initiated by the NSF called for a Software Sharing Plan as have a number of NIH grants (See the National Science Foundation Grant Solicitation, “Core Techniques and Technologies for Advancing Big Data Science & Engineering (BIGDATA),” http://www.nsf.gov/pubs/2012/nsf12499/nsf12499.htm).

In addition to funding agency or institutional requirements, journals exert a tremendous amount of influence on communication standards for scientific knowledge dissemination. We follow up on one principle and one recommendation made in a 2003 National Academies report [16] stating:

**Principle 1. Authors should include in their publications the data, algorithms, or other information that is central or integral to the publication–that is, whatever is necessary to support the major claims of the paper and would enable one skilled in the art to verify or replicate the claims.**

This is a *quid pro quo*–in exchange for the credit and acknowledgement that come with publishing in a peer-reviewed journal, authors are expected to provide the information essential to their published findings. (p. 5)

**Recommendation 6. Scientific journals should clearly and prominently state (in the instructions for authors and on their Web sites) their policies for distribution of publication-related materials, data, and other information. Policies for sharing materials should include requirements for depositing materials in an appropriate repository. Policies for data sharing should include requirements for deposition of complex datasets in appropriate databases and for the sharing of software and algorithms integral to the findings being reported. The policies should also clearly state the consequences for authors who do not adhere to the policies and the procedure for registering complaints about noncompliance.**

Many journals do not specify policies about sharing data and materials in their instructions to authors. By incorporating transparent standards into their official policies (including a statement of consequences for authors who do not comply), journals can encourage compliance. (p. 10).

Principle 1 calls for the dissemination of data, software, and all information necessary for a researcher to “verify or replicate the claims” made in the publication. Recommendation 6 is a call for journals to clarify and explicitly state their policies regarding data and code release requirements, and to state consequences for authors who do not comply with these requirements. Appendix S1 lists the Principles and Recommendations given by the National Academies task force in their entirety. The two highest ranked journals in scientific publication, *Nature* and *Science*, both now require authors to make available the data underlying their published results upon request, and in February of 2011 *Science* extended this policy to include code and software [13]. One fundamental research question we seek to address is the role of leadership in journal policy setting, specifically whether this action on the part of flagship journals could be expected to create a “trickle down” effect to other journals.

## Methods

We chose to include the journals classified in the ISI Web of Knowledge journal categorizations “Mathematical & Computational Biology,” “Statistics & Probability,” and “Multidisciplinary Sciences” since they are likely to report computational results and therefore likely to be composed of journals developing data and code sharing policies. We chose to include computational biology because of the strides made toward data sharing in this field over the last decade or so [17], [18]. We then chose to add 5 additional journals, due to their high impact factors and likelihood of publishing computational results (Nature Genetics, Cell, Lancet, Nature Physics, and Materials Science and Engineering Reports). After removing the handful of journals that have ceased active publication, this effort selected 170 journal titles as shown in Table 2 (Appendix S2 gives the complete alphabetical list of journal titles included in this study).

**Table 2:** ISI Classifications Represented in the Journal Titles.

ISI Classification	Count
Statistics & Probability	98
Multidisciplinary Science	45
Mathematical & Computational Biology	30
Genetics & Heredity (Nature Genetics)	1
Biochemistry & Molecular Biology; Cell Biology (Cell)	1
Medicine, General & Internal (Lancet)	1
Physics, Multidisciplinary (Nature Physics)	1
Materials Science, Multidisciplinary; Physics, Applied (Materials Science & Engineering R - Reports)	1
In both “Statistics & Probability” and “Mathematical & Computational Biology”	−8
**Adjusted Total**	**170**

Tables 3 and 4 show the distribution of impact factors for the journal titles and the publishing houses represented. Unsurprisingly, given the distribution of impact factor rankings across journals in general, a majority of the titles in this study have an impact factor of 1 or less (110 of 170) and 15 titles have an impact factor of 5 or greater. As shown in Table 5, the Springer Publishing House publishes the greatest percent of the journal titles in this study at 17.1%, and about half the journals are published by one of Springer, Wiley, Elsevier, and Taylor & Francis.

**Table 3:** Distribution of Impact Factors for Journal Titles.

ISI Impact Factor	Count
30–35	5
10–29	2
8–9	1
6–7	1
4–5	6
2–3	45
0–1	110
**Total**	**170**

**Table 4:** Publishing Houses for Journal Titles.

Publishing House	Count	Percent
Springer (incl. Springer Heidelberg, Springer/Plenum Publishers, MAIK Nauka Interperiodica Springer, BioMed Central)	29	17.1%
Wiley (incl. John Wiley & Sons, Wiley-Blackwell Publishing, and Wiley-VCH Verlag GmbH)	20	11.8%
Reed Elsevier (incl. Elsevier Science BV, Academic Press LTD – Elsevier Science, and Pergamon-Elsevier Science LTD)	19	11.2%
Taylor & Francis (incl. Lawrence Erlbaum Associates Inc. and Routledge Journals)	13	7.6%
Macmillan (Nature Publishing Group)	3	1.8%
Scientific Societies	31	18.2%
Other For-Profit Publishers	33	19.4%
Not-For-Profit Non-Society Publishers	22	12.9%
**Total**	**170**	**100%**

**Table 5:** Classification of Journal Policies.

Data Sharing, Code Sharing, and Supplemental Materials Policies
1. Required as condition of publication, certain exceptions permitted (e.g. preserving confidentiality of human subjects)
2. Required but may not affect editorial/publication decisions
3. Explicitly encouraged/addressed; may be reviewed and/or hosted
4. Implied
5. No mention

We inspected the websites of all of these journal titles, once in June of 2011 and a second time in June of 2012, to ascertain their policies on data sharing, code sharing, and supplementary materials. Journal policies typically did not explicitly define “code,” nor did they make a distinction between commercial code and open source code. *Science*, for example, states that “All computer codes involved in the creation or analysis of data must also be available to any reader of *Science*. … Any restrictions on the availability of data, codes, or materials, including fees and original data obtained from other sources … must be disclosed to the editors upon submission.” [13]. Each journal policy was evaluated on a 5-point scale, as shown in Table 6. We included supplemental materials policy in our investigation as a proxy for openness and as a possible bellwether for changes in data and code policies. Supplemental materials, however, tend to include figures and explanations that were not included in the main article rather than data or code.

**Table 6:** Net Changes in Journal Policy Classifications from 2011 to 2012.

Data Sharing Policy (n = 170)	2011	2012	Change
Required as condition of publication, barring exceptions	18	19	1
Required but may not affect editorial decisions	3	10	7
Explicitly encouraged/addressed, may be reviewed and/or hosted	35	30	−5
Implied	0	5	5
No mention	114	106	−8
**Code Sharing Policy (n = 170)**	**2011**	**2012**	**Change**
Required as condition of publication, barring exceptions	6	6	0
Required but may not affect editorial decisions	6	6	0
Explicitly encouraged/addressed, may be reviewed and/or hosted	17	21	4
Implied	0	3	3
No mention	141	134	−7
**Supplemental Materials Policy (n = 170)**	**2011**	**2012**	**Change**
Required as condition of publication, barring exceptions	8	6	−2
Required but may not affect editorial decisions	7	10	3
Explicitly encouraged/addressed, may be reviewed and/or hosted	86	93	7
Implied	4	3	−1
No mention	65	58	−7

We also collected information to supplement these rankings to help illuminate and contrast policies. Each data, code, and supplemental materials policy ranking was augmented depending on whether they were specified to be shared via submission to the journal, upon request from readers, or whether this was left unspecified. The policy ranking was further augmented to indicate whether the journal specified that the author was intended to share with colleagues and other researchers in the field, or with the general public (i.e. unspecified). The code sharing policy classification was augmented with an additional parameter that signaled whether the journal has restricted the code policy to apply only to articles with “substantial” code or software. We also recorded whether the journal explicitly permitted either the posting of the final version or a draft of the published version of the article on authors’ website. The final factor we recorded was whether the journal indicated it would review data, code, or supplemental materials submissions, and whether these would be hosted by the journals. These additional policy classifications captured the vast majority of journal policy variation.

## Results

Classifying journal policies according to the ranking system given in Table 5 yields a snapshot of the journal publication standards on the availability of the data and code associated with published computational findings. The data from this study are available at http://www.stodden.net/JournalPolicies2013/. Table 6 gives counts for each classification by year, including the change from 2011 to 2012.

The majority of journal titles included in our study have not followed the recommendations of the National Academies committee mentioned in the introduction by describing their data and code sharing policies on their websites. In June of 2012, 62% of the journals in this study make no mention of a data policy and 79% make no mention of a software policy. However, 66% have a supplemental materials policy. Of the remaining journals that mention data or software policies on their website, the majority encourage the practice of sharing but do not require it: 47% of journals with a data sharing policy encourage sharing and 45% require it (including the 16% who state noncompliance with this requirement will not affect publication decisions). Similarly, 56% of journals with a software policy choose encouragement of code sharing, and 32% require code disclosure (including the 16% who indicate that noncompliance with this requirement will not affect publication decisions). Supplemental materials exhibited a different pattern. From 2011 to 2012 there was a net reduction of two journals requiring supplemental materials, and there was a net gain of seven journals adopting explicit encouragement of supplemental materials inclusion with publication.

Overall, 30 journals made a data policy change from 2011 to 2012, 12 made a change in their software policy, and 36 made a change to their supplemental data policy. The net change numbers in Table 6 indicate this is markedly in the direction of openness. There are a total of 104 journals with neither an open data nor open code policy in 2012, down from 110 in 2011. In other words, 39% of journals had some form of open data or open code policy in 2012, up from 35% in 2011.

Only three journals in 2012 included a qualifier in their code release policy by stating it applied only to articles that employed substantial software. In 2012 eleven journals planned to review supplemental materials submissions, and 69 were explicitly willing to host supplemental materials. Also in 2012, five journals would review data submissions and 10 were willing to host such submissions, whereas two journals would review code, and two would host code (these were not the same two journals). Table 7 summarizes these findings. Most journals with a supplemental materials policy were willing to host submissions, and of those with a data sharing policy nearly 16% were willing to host submitted data.

**Table 7:** Journal Review and Hosting Policies, 2012.

Data Sharing Policy (n = 64)	2012 Count	Percent of Total
Reviewed	5	7.8%
Hosted	10	15.6%
**Code Sharing Policy (n = 36)**		
Reviewed	2	5.6%
Hosted	2	5.6%
**Supplemental Materials Policy (n = 112)**		
Reviewed	11	9.8%
Hosted	69	61.6%

**Figure 1:**
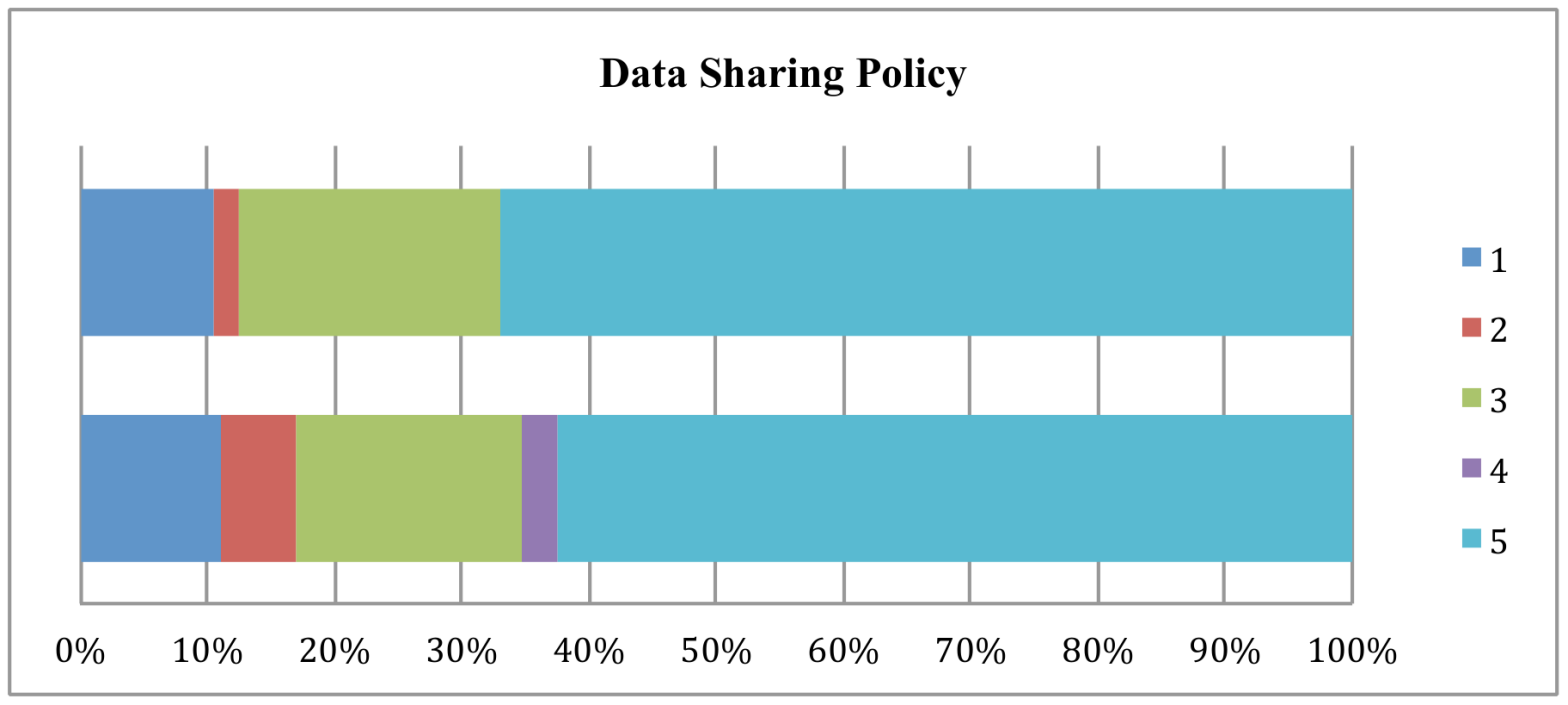
Changes in Data Sharing Policy, 2011–2012.

Figures 1 through 3 show the changes from 2011 to 2012 in how journal policy intends the data, code, or supplemental materials to be accessed. The most important finding is that there has been a shift away from journals accepting data to policies that provide for reader access upon request to the authors.

**Figure 2:**
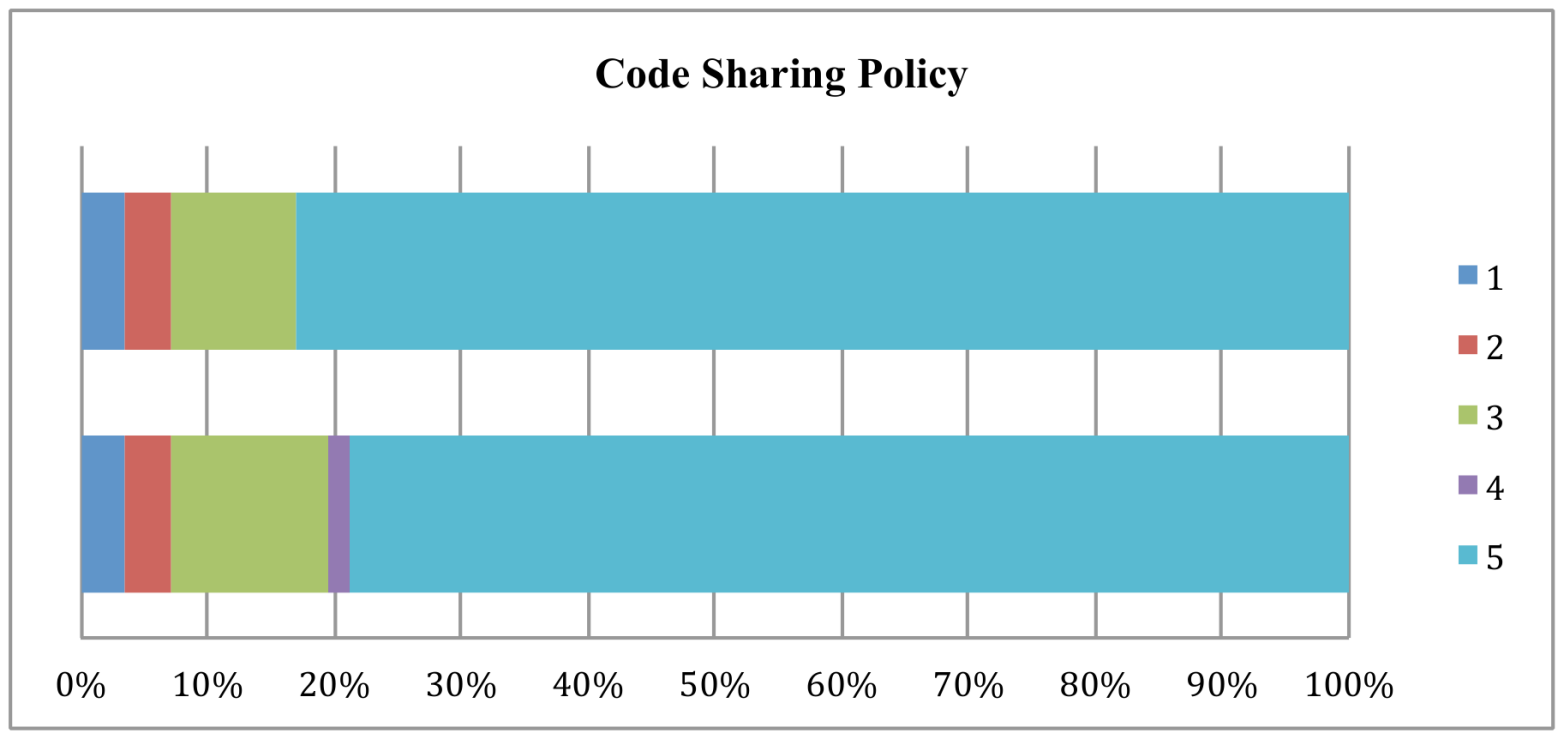
Changes in Code Sharing Policy, 2011–2012.

**Figure 3:**
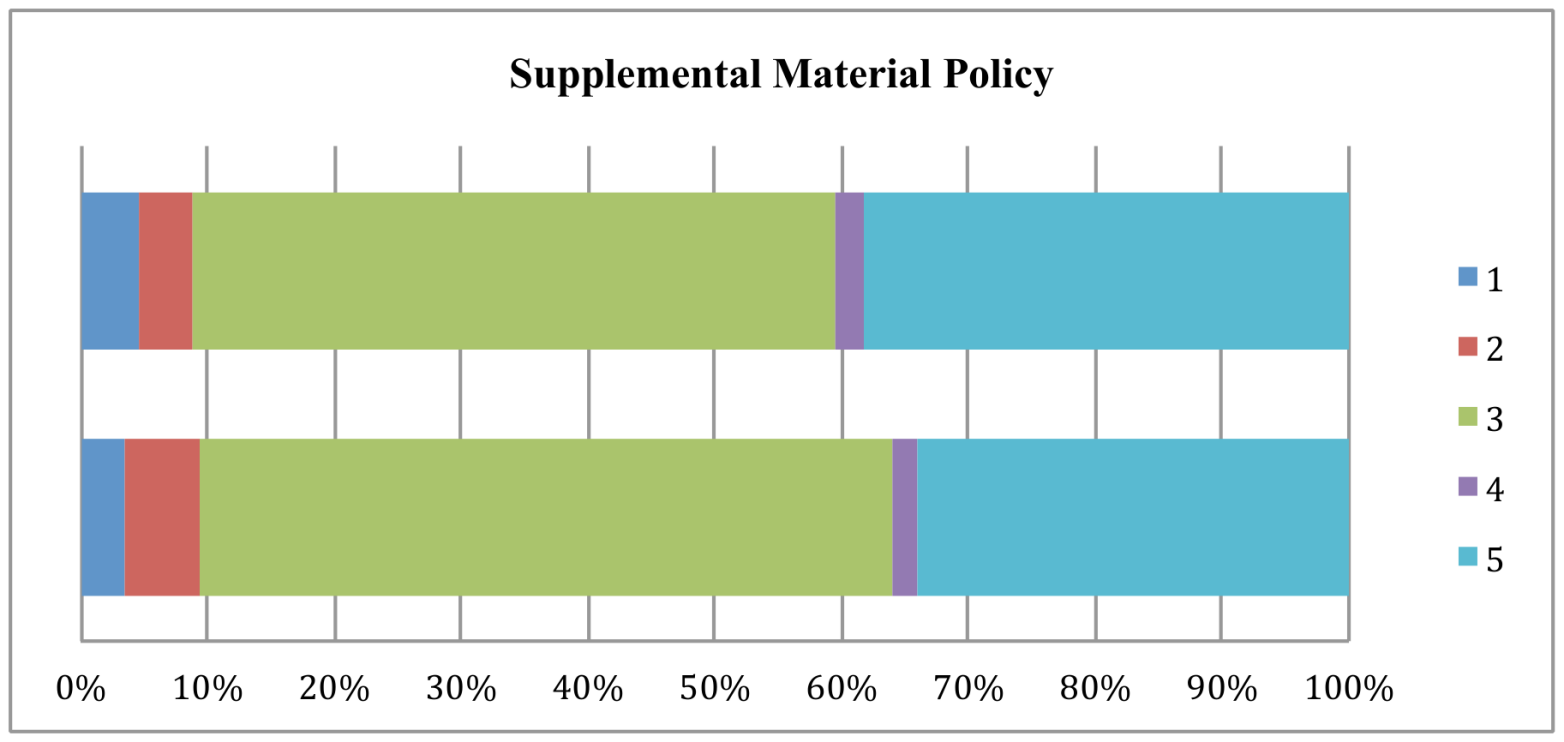
Changes in Supplemental Materials Policy, 2011–2012.

The higher impact journals appear to be adopting open data and code requirements more readily than lower impact journals, possibly indicating that higher impact journals are more comfortable increasing the demands on contributing authors. We were also interested in whether journal ownership has an influence on whether or not a particular journal has a data or code sharing policy. We therefore used a logistic model to regress impact factor and publisher data on a binary variable indicating the presence of a data or code sharing policy (leaving the “Not-For-Profit Publisher” variable out). We give the coefficient estimates in Table 8. At the 5% level all of Elsevier, Wiley, and Scientific Society Publisher have significant coefficient estimates, indicating that having one of these publishers increases the log odds of a data or code sharing policy (for Elsevier and Wiley the log odds roughly doubles, and if a Scientific Society publishes then the log odds of having a data or code policy increase by about 1.7). Impact Factor is the most highly significant determinant of the existence of a journal data or code sharing policy, indicating the tendency of the high impact journals to lead policy changes that create extra encumbrances for authors. Since Macmillan, publisher of the Nature family of journals, has three observations in this dataset the associated large standard error is unsurprising.

**Table 8:** Regression Coefficients from Predicting Open Data and Code Policies by Publisher and Impact Factor.

Variable	CoefficientEstimate	Std Error	p-value
Intercept	−2.4600	0.7207	0.0006
Impact Factor	0.5271	0.1719	0.0022
Elsevier	2.0601	0.8342	0.0135
Taylor & Francis	0.2721	1.0225	0.7902
Macmillan	9.0718	980.7362	0.9926
Springer	0.3760	0.8046	0.6403
Wiley	1.9021	0.8011	0.0176
Scientific Society Publisher	1.6794	0.7529	0.0257
Other For-Profit Publisher	1.2880	0.7594	0.0899

Of the 13 journals that had a change to their code policy, seven were new policies instantiated sometime between June 2011 and June 2012. Of these seven, all were journals that had pre-existing data policies in place in 2011. It seems code disclosure policies follow on the heels of data disclosure policies in this sample of journals. For the seven journals that adopted new code disclosure policies, four of them also shifted their data disclosure requirements from encouraged to required (the others had no change to their existing data policies). This “follow-on” hypothesis is also supported by the greater proportion of journals with a data access policy versus those with a code access policy in 2012, 38% and 21% respectively. Data policy appears to be the gateway toward more open policies generally. Supplemental materials policies seem to lead or be in tandem with data policies. Of the 11 new data policies implemented from 2011 to 2012, 5 of those journals had no supplemental materials policy in 2011, but by 2012 nine of the 11 journals with new data sharing policies had supplemental materials policies.

We also ranked the 170 journals in our study regarding their policies about open access to the published paper itself. We were especially interested in whether there was a correlation between journals that are amenable to open access and the availability of data and code through journal policy. We were also interested in whether a “follow-on” effect existed for scholarly object policies, i.e. data, code, and supplementary materials, from open access policy. We saw very little change however in open access policies for our 170 journals from 2011 to 2012. Table 9 details these differences: the net change was an increase in one journal requiring open access publication, one journal shifting away from delayed open access or membership requirements, a net increase of one journal explicitly permitting the posting of the draft or final versions of the paper on the web, and finally a net decrease of one journal requiring subscription.

**Table 9:** Changes in Open Access Policy 2011–2012.

Open Access Policy (n = 170)	2011	2012	Change
Open access	29	30	1
Open access with delay and/or journal membership requirement	73	72	−1
Subscription but authors explicitly allowed to post draft or final	13	14	1
Subscription	55	54	−1

With such small changes to open access policy from 2011 to 2012, changes in open access policy do not appear to be driving changes in data and code sharing policies. We examined the correlation between open access policies and data and code sharing policies as follows. We divided the journals into open access (2012 classifications 1 and 2 from Table 5) and subscription (2012 classifications 3 and 4) and examined at the differences in 2012 data and code policies for the two groups. If open access journals are more likely to adopt open data and code policies the following year, we should see significant differences between the two groups in 2012. Table 10 shows these differences. Although the proportion of open access journals with a data or code policy is greater than the proportion for subscription journals, a chi-square test of independence is not significant for these data (p = 0.44). Therefore these data provide no evidence that an open access policy indicates a greater likelihood of an open data or code policy. At present, access to published papers appears to be a separate issue to reproducible research from the journal perspective.

**Table 10:** Open Access and Open Data/Code Policies 2012.

Publication Access	Data or Code Policy	No Mention	Total
Open Access	42	60	102
Subscription	24	44	66
**Total**	66	104	170

## Discussion

The overall move toward data and code availability by journals is clear. Of the journals that had a change in their data sharing policy from 2011 to 2012, eleven adopted a data policy for the first time, a net of five journals shifted from encouraging data sharing to requiring it, while four dropped their data access policy. The two remaining journals with a change shifted to data sharing being implied and to explicitly stating a failure to share data will not affect editorial decisions such as publication, respectively. This shows a marked shift toward more stringent data sharing policies, in the course of only one year.

With only two years of data it is difficult to speculate as to the reasons for the increases in the number of journals with code and data access policies, but there are some exogenous policy changes that may be affecting journal policy creation. As mentioned previously, in January of 2011 the National Science Foundation began requiring all grant application to include a two-page Data Management Plan, describing the intended availability and archiving of any dataset produced in the course of the research. The genomics research community was rocked by flawed cancer research emerging from researchers at Duke University [19], [20], which culminated in the Institute of Medicine report entitled “Evolution of Translational Omics: Lessons Learned and the Path Forward” recommending, among other things, code and data disclosure for biomarker tests seeking FDA approval to proceed to clinical trial [21].

One measure that may help shed light on the rationale underlying the shift to data and code disclosure policies is the wording in the policy statement. We examined the frequency of the use of the term “reproducibility” or similar terms such as “replication,” in journal policy statements regarding data and code. We found that eleven journals of the 66 with either a data sharing or code sharing policy in 2012 specifically referenced these terms when explaining their publication policies, whereas only four journals did so in 2011 (the eleven include those four, no journal that mentioned reproducibility as a rationale for its policies stopped doing so in 2012). This seems to indicate the importance of reproducibility as a rationale underlying data and code access policies. Of the eleven mentioning reproducible research terms in their policies, all except two had open data policies (six required and three encouraged), and five had required code disclosure policies (one encouraged code disclosure). Of course if a journal does not explicitly mention these terms, that does not preclude reproducibility from being their underlying rationale for implementing data and code access policies. The average impact factor for this group of eleven mentioning reproducibility was 10, much higher than the average impact factor for all journals in this study of 1.82. In fact, the two journals in this group of eleven that do not have data policies have the lowest impacts factors of the group at 0.324 and 0.554.

### Conclusions and Future Work

In this paper we sought a unified understanding of the evolution of journal policy. We studied the data and code sharing policies of a group of computational journals in June of 2011 and then again in June of 2012. We documented 170 journal policies, classifying their data, code, supplemental materials, and open access policies. We hypothesized that open data and code policies are in the process of being adopted more widely, that data policies would lead code policies, and that open access journals would be more likely to have policies making data and code open as well.

We found evidence to support our first two hypotheses, and little evidence to support the third. In June of 2012, 38% of the journals in this study had a data policy, 22% had a code policy, and 66% had a supplemental materials policy. This is an increase from June of 2011 when the proportions were 33%, 17%, and 62% respectively. Of journals that have open data and code policies, they tend to adopt open data policies first followed by open code policies. Perhaps surprisingly, supplemental materials policies do not seem to lead data or code polices in a similar way, nor do they appear to crowd out or displace data and code policies. There seems to be no difference in open data and code policy adoption rates for open access versus subscription journals.

This study was limited to the journals listed in the following three ISI Web of Knowledge classifications: “Mathematical & Computational Biology,” “Statistics & Probability,” and “Multidisciplinary Sciences” as well as a handful of additional journals. This selection has a bias toward bioinformatics and life sciences research due to the inclusion of computational biology journals. The Protein Data Bank (PDB) for example was established in 1971 and deposit within PDB is required for papers describing three-dimensional structures of biological macromolecules [22]. The Human Genome Project promulgated widely agreed upon community standards of data sharing as early as 1996 that established data openness as a norm in the field of genomics [23]. This long history is the exception in data intensive empirical science and was part of the rationale behind including computational biology journals in the study, in order to understand a more mature and more pervasive response to the question of open data. Future work however could expand the sample under study to include other computational fields. Such an expansion would reduce potential bias due to the inclusion of computational biology journals and verify whether the same patterns of policy adoption persist in other areas.

An open question in this study is why several journals reduced or eliminated their data and code sharing requirements from 2011 to 2012. It would be instructive to learn on a case by case basis why this occurred. This would provide information about which policies seem to work best for which fields.

This study models open data and code policy adoption, using impact factor and publishing house as explanatory variables, but research could be carried out using a more extensive set of confounding variables such as field characteristics, journal size, journal age, frequency of publication, proportion of computational results published in the journal, proportion of computational results publishing in the field. In this research we also introduce a novel dataset on journal policy.

This study does not take into account the enforcement and effectiveness of data and code sharing policies enacted by journals. It documents the state of such policies on journal websites in 2011 and 2012, but does not extend the analysis to effectiveness. An important question is whether the existence of such policies as described in this study materially affects the ability to access the data and code that underlies published computational results.

This study shows that ten years after the publication of the National Academies report “Sharing Publication-Related Data and Materials: Responsibilities of Authorship in the Life Sciences” [16], progress is being made regarding the sharing of data and code that underlie research publications. In particular, we focus on the rapid growth in the number of journal policies on research data and code, with a one year increase to 2012 of 16% in journals with data policies and a 30% increase in those with code policies. We are, however, still far from the vision of the NAS report, especially Recommendation 6, which calls for scientific journals to “state their policies for distribution of publication-related materials, data, and other information,” which should include “requirements for depositing materials in an appropriate repository”… and for “the sharing of software and algorithms integral to the findings being reported.” In 2012, 38% of the journals in this study had a data policy and 22% had a code policy. A wider recognition of the importance of policies that support reproducible computational research is imperative.

## Supporting Information

Appendix S1 **Principles and Recommendations from the National Academies 2003 book “Sharing Publication-Related Data and Materials: Responsibilities of Authorship in the Life Sciences.”**
(DOCX)

Appendix S2 **Journal Titles.**
(DOCX)

Dataset S1 **Journal Policies Dataset.**
(XLSX)
