# Supplementary material for: Toward Reproducible Computational Research: An Empirical Analysis of Data and Code Policy Adoption by Journals
Source: PLoS One. 2013 Jun 21;8(6):e67111. doi: 10.1371/journal.pone.0067111 (PMC3689732; doi:10.1371/journal.pone.0067111)
Supplement: Appendix S1 — Principles and Recommendations from the National Academies 2003 book “Sharing Publication-Related Data and Materials: Responsibilities of Authorship in the Life Sciences.” (DOCX) [file pone.0067111.s001.docx]

**Appendix 1: Principles and Recommendations from the National Academies 2003 book “Sharing Publication-Related Data and Materials: Responsibilities of Authorship in the Life Sciences”**

Excerpted Principles and Recommendations from the National Academies 2003 book, “Sharing Publication-Related Data and Materials: Responsibilities of Authorship in the Life Sciences” pages 4-14.

Reprinted with permission from the National Academy of Sciences, Courtesy of the

National Academies Press, Washington, D.C.

See <http://www.nap.edu/catalog.php?record_id=10613> for the full exposition.

UPSIDE

“the uniform principle for sharing integral data and materials expeditiously (UPSIDE):” **Community standards for sharing publication-related data and materials should flow from the general principle that the publication of scientific information is intended to move science forward. More specifically, the act of publishing is a *quid pro quo* in which authors receive credit and acknowledgment in exchange for disclosure of their scientific findings. An author’s obligation is not only to release data and materials to enable others to verify or replicate published findings (as journals already implicitly or explicitly require) but also to provide them in a form on which other scientists can build with further research. All members of the scientific community—whether working in academia, government, or a commercial enterprise—have equal responsibility for upholding community standards as participants in the publication system, and all should be equally able to derive benefits from it.**

DATA AND SOFTWARE

**Principle 1. (Chapter 3) Authors should include in their publications the data, algorithms, or other information that is central or integral to the publication—that is, whatever is necessary to support the major claims of the paper and would enable one skilled in the art to verify or replicate the claims.**

**Principle 2. (Chapter 3) If central or integral information cannot be included in the publication for practical reasons (for example, because a dataset is too large), it should be made freely (without restriction on its use for research purposes and at no cost) and readily accessible through other means (for example, on-line). Moreover, when necessary to enable further research, integral information should be made available in a form that enables it to be manipulated, analyzed, and combined with other scientific data.**

**Principle 3. (Chapter 3) If publicly accessible repositories for data have been agreed on by a community of researchers and are in general use, the relevant data should be deposited in one of these repositories by the time of publication.**

MATERIALS

**Principle 4. (Chapter 4) Authors of scientific publications should anticipate which materials integral to their publications are likely to be requested and should state in the “Materials and Methods” section or elsewhere how to obtain them.**

**Principle 5. (Chapter 4) If a material integral to a publication is patented, the provider of the material should make the material available under a license for research use.**

RECOMMENDATIONS

**Recommendation 1. (Chapter 3) The scientific community should continue to be involved in crafting appropriate terms of any legislation that provides additional database protection.**

**Recommendation 2. (Chapter 4) It is appropriate for scientific reviewers of a paper submitted for publication to help identify materials that are integral to the publication and likely to be requested by others and to point out cases in which authors need to provide additional instructions on obtaining them.**

**Recommendation 3. (Chapter 4) It is not acceptable for the provider of a publication-related material to demand an exclusive license to commercialize a new substance that a recipient makes with the provider’s material or to require collaboration or coauthorship of future publications.**

**Recommendation 4. (Chapter 4) The merits of adopting a standard MTA should be examined closely by all institutions engaged in technology transfer, and efforts to streamline the process should be championed at the highest levels of universities, private research centers, and commercial enterprises.**

**Recommendation 5. (Chapter 4) As a best practice, participants in the publication process should commit to a limit of 60 days to complete the negotiation of publication-related MTAs and transmit the requested materials or data.**

**Recommendation 6. (Chapter 6) Scientific journals should clearly and prominently state (in the instructions for authors and on their Web sites) their policies for distribution of publication-related materials, data, and other information. Policies for sharing materials should include requirements for depositing materials in an appropriate repository. Policies for data sharing should include requirements for deposition of complex datasets in appropriate databases and for the sharing of software and algorithms integral to the findings being reported. The policies should also clearly state the consequences for authors who do not adhere to the policies and the procedure for registering complaints about noncompliance.**

**Recommendation 7. (Chapter 6) Sponsors of research and research institutions should clearly and prominently state their policies for distribution of publication-related materials and data by their grant or contract recipients or employees.**

**Recommendation 8. (Chapter 6) If an author does not comply with a request for data or materials in a reasonable time period (60 days) and the requestor has contacted the author to determine if extenuating circumstances (travel, sabbatical, or other reasons) may have caused the delay, it is acceptable for the requestor to contact the journal in which the paper was published. If that course of action is not successful in due course (another 30 days), the requestor may reasonably contact the author’s university or other institution or the funder of the research in question for assistance. Those entities should have a policy and process in place for responding to such requests for assistance in obtaining publication-related data or materials.**

**Recommendation 9. (Chapter 6) Funding organizations should provide the recipients of research grants and contracts with the financial resources needed to support dissemination of publication-related data and materials.**

**Recommendation 10. (Chapter 6) Authors who have received data or materials from other investigators should acknowledge such contributions appropriately.**

FINAL STATEMENT

**Universal adherence, without exception, to a principle of full disclosure and unrestricted access to data and materials that are central or integral to published findings will promote cooperation and prevent divisiveness in the scientific community, maintain the value and prestige of publication, and promote the progress of science.**
