# Supplementary material for: Toward Reproducible Computational Research: An Empirical Analysis of Data and Code Policy Adoption by Journals
Source: PLoS One. 2013 Jun 21;8(6):e67111. doi: 10.1371/journal.pone.0067111 (PMC3689732; doi:10.1371/journal.pone.0067111)
Supplement: Appendix S2 — Journal Titles. (DOCX) [file pone.0067111.s002.docx]

**Appendix 2: Journal Titles**

These journal titles were obtained from the ISI Web of Science 2011 classifications of “Mathematical & Computational Biology,” “Statistics & Probability,” and “Multidisciplinary Sciences,” and include five additional titles.

Acta Scientiarum - Technology

Advances in Applied Probability

Advances in Complex Systems

Algorithms for Molecular Biology

American Scientist

American Statistician

Anais da Academia Brasileira de Ciências

Annals of Applied Probability

Annals of Applied Statistics

Annales de l'Institut Henri Poincaré, Probabilités et Statistiques

Annals of the Institute of Statistical Mathematics

Annals of the New York Academy of Sciences

Annals of Probability

Annals of Statistics

Applied Stochastic Models in Business and Industry

Arab Gulf Journal of Scientific Research

Arabian Journal for Science and Engineering

Advances in Statistical Analysis

ASTIN Bulletin

Australian & New Zealand Journal of Statistics

Bulletin of Mathematical Biology

Bayesian Analysis

Bernoulli

Bioinformatics

Biometrical Journal

Biometrics

Biometrika

Biostatistics

BMC Bioinformatics

BMC Systems Biology

British Journal of Mathematical & Statistical Psychology

Canadian Journal of Statistics

Cell

Chemometrics and Intelligent Laboratory Systems

Chinese Science Bulletin

Combinatorics, Probability & Computing

Communications in Statistics - Simulation and Computation

Communications in Statistics - Theory and Methods

Complexity

Computers in Biology and Medicine

Computational Statistics & Data Analysis

Computational Statistics

Comptes rendus de l'Academie bulgare des Sciences

Current Bioinformatics

Current Science

Defence Science Journal

Discrete Dynamics in Nature and Society

Econometrics Journal

Econometric Reviews

Econometrica

Electronic Communications in Probability

Electronic Journal of Probability

Endeavour

Environmental and Ecological Statistics

Environmetrics

Evolutionary Bioinformatics

Finance and Stochastics

Fractals - Complex Geometry Patterns and Scaling in Nature and Society

Fuzzy Sets and Systems

Hacettepe Journal of Mathematics and Statistics

Herald of the Russian Academy of Sciences

IBM Journal of Research and Development

IEEE-ACM Transactions on Computational Biology and Bioinformatics

IEEE Transactions on Information Technology in Biomedicine

IET Systems Biology

Infinite Dimensional Analysis, Quantum Probability, and Related Topics

Insurance Mathematics & Economics

International Journal of Agricultural and Statistical Sciences

International Journal of Bifurcation and Chaos

International Journal of Data Mining and Bioinformatics

International Journal of Game Theory

International Journal of Physical Sciences

International Statistical Review

Interdisciplinary Science Reviews

Iranian Journal of Science and Technology Transcation A - Science

Issues in Science and Technology

Journal of Agricultural, Biological, and Environmental Statistics

Journal of the American Statistical Association

Journal of Applied Probability

Journal of Applied Statistics

Journal of Biological Systems

Journal of Biopharmaceutical Statistics

Journal of Business & Economic Statistics

Journal of Chemometrics

Journal of Computational Biology

Journal of Computational and Graphical Statistics

Journal of Computational Neuroscience

Johns Hopkins APL Technical Digest

Journal of the Korean Statistical Society

Journal of Mathematical Biology

Journal of Molecular Graphics & Modelling

Journal of Multivariate Analysis

Journal of Nonparametric Statistics

Journal of Quality Technology

Journal of the Royal Society Interface

Journal of the Royal Statistical Society Series A - Statistics in Society

Journal of the Royal Statistical Society Series B - Statistical Methodology

Journal of the Royal Statistical Society Series C - Applied Statistics

Journal of the Royal Society of New Zealand

Journal of Statistical Computation and Simulation

Journal of Statistical Planning and Inference

Journal of Statistical Software

Journal of Theoretical Biology

Journal of Theoretical Probability

Journal of Time Series Analysis

Kuwait Journal of Science & Engineering

Lancet

Lifetime Data Analysis

Maejo International Journal of Science and Technology

Materials Science & Engineering R - Reports

Mathematical Biosciences

Mathematical Medicine and Biology - a Journal of the IMA

Mathematical Population Studies

Medical & Biological Engineering & Computing

Methodology and Computing in Applied Probability

Metrika

Multivariate Behavioral Research

Nature Genetics

Nature Physics

Nature

Naturwissenschaften

Open Systems & Information Dynamics

Oxford Bulletin of Economics and Statistics

Proceedings of the Estonian Academy of Sciences

Proceedings of the Japan Academy Series B - Physical and Biological Sciences

Proceedings of the National Academy of Sciences of India Section A - Physical Sciences

Proceedings of the National Academy of Sciences of the United States of America

Proceedings of the Romanian Academy Series A - Mathematics, Physics, Technical Sciences, Information Science

Proceedings of the Royal Society A - Mathematical, Physical, and Engineering Sciences

Pharmaceutical Statistics

Philosophical Transactions of the Royal Society A - Mathematical, Physical, and Engineering Sciences

PLoS Computational Biology

Probability in the Engineering and Informational Sciences

Probability Theory and Related Fields

Probabilistic Engineering Mechanics

Progress in Natural Science

Quality & Quantity

R&D Magazine

South African Journal of Science

SAR & QSAR in Environmental Research

Scandinavian Journal of Statistics

Scientific American

Science and Engineering Ethics

Scientific Research and Essays

Science

Statistics and Operations Research Transactions

Journal: Statistical Applications in Genetics and Molecular Biology

Statistics and Computing

Statistics in Medicine

Statistical Methods and Applications

Statistical Methods in Medical Research

Statistical Modelling

Statistica Neerlandica

Statistical Papers

Statistics & Probability Letters

Statistical Science

Statistica Sinica

Stata Journal

Statistics

Stochastic Analysis and Applications

Stochastics and Dynamics

Stochastic Environmental Research and Risk Assessment

Stochastic Models

Stochastic Processes and Their Applications

Survey Methodology

Transactions of the Royal Society of South Australia

Technometrics

TEST

Theory of Probability and its Applications

The Scientific World Journal
